# 4-Benzene­sulfonamido­benzoic acid

**DOI:** 10.1107/S1600536809047291

**Published:** 2009-11-14

**Authors:** Hafiz Muhammad Adeel Sharif, Gui-Ying Dong, Muhammad Nadeem Arshad, Islam Ullah Khan

**Affiliations:** a Materials Chemistry Laboratory, Department of Chemistry, Government College University, Lahore 54000, Pakistan; b College of Chemical Engineering and Biotechnology, Hebei Polytechnic University, Tangshan 063009, People’s Republic of China

## Abstract

In the mol­ecule of the title sulfonamide compound, C_13_H_11_NO_4_S, the dihedral angle between the planes of the benzene ring and the carboxyl substituent group is 6.7 (4)°. The two aromatic rings are inclined at 45.36 (15)° to one another. In the crystal, adjacent mol­ecules are linked *via* classical inter­molecular N—H⋯O and O—H⋯O, and non-classical C—H⋯O hydrogen bonds, which stabilize the crystal structure.

## Related literature

For the biological activity and pharmaceutical applications of sulfonamide derivatives, see: Innocenti *et al.* (2004 ▶); Parai *et al.* (2008 ▶); Rathish *et al.* (2009 ▶); Selvam *et al.* (2001 ▶). For related structures of sulfonamide derivatives with 4–amino­benzoic acid, see: Arshad *et al.* (2009 ▶); Khan *et al.* (2009 ▶); Nan & Xing (2006 ▶).
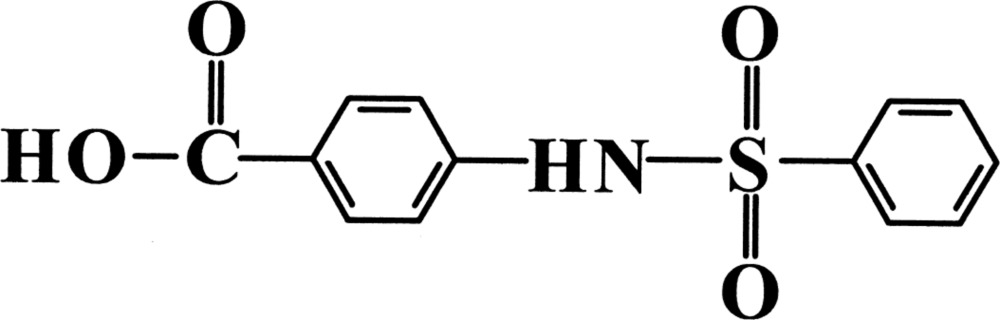


## Experimental

### 

#### Crystal data

C_13_H_11_NO_4_S
*M* *_r_* = 277.30
Monoclinic, 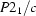

*a* = 5.2050 (3) Å
*b* = 37.726 (2) Å
*c* = 7.3781 (4) Å
β = 117.510 (3)°
*V* = 1284.98 (13) Å^3^
*Z* = 4
Mo *K*α radiation
μ = 0.26 mm^−1^
*T* = 295 K
0.26 × 0.21 × 0.19 mm

#### Data collection

Bruker CCD diffractometer
Absorption correction: multi-scan (**SADABS**; Sheldrick, 1996 ▶) *T*_min_ = 0.935, *T*_max_ = 0.958
13550 measured reflections
3185 independent reflections
2633 reflections with *I* > 2σ(*I*)
*R*_int_ = 0.025

#### Refinement

*R*[*F* ^2^ > 2σ(*F* ^2^)] = 0.065
*wR*(*F* ^2^) = 0.199
*S* = 1.10
3185 reflections
172 parameters
H-atom parameters constrained
Δρ_max_ = 0.38 e Å^−3^
Δρ_min_ = −0.36 e Å^−3^

### 

Data collection: *SMART* (Bruker, 1998 ▶); cell refinement: *SAINT* (Bruker, 1999 ▶); data reduction: *SAINT*; program(s) used to solve structure: *SHELXS97* (Sheldrick, 2008 ▶); program(s) used to refine structure: *SHELXL97* (Sheldrick, 2008 ▶); molecular graphics: *SHELXTL* (Sheldrick, 2008 ▶); software used to prepare material for publication: *SHELXTL*.

## Supplementary Material

Crystal structure: contains datablocks I, global. DOI: 10.1107/S1600536809047291/rk2179sup1.cif

Structure factors: contains datablocks I. DOI: 10.1107/S1600536809047291/rk2179Isup2.hkl

Additional supplementary materials:  crystallographic information; 3D view; checkCIF report

## Figures and Tables

**Table 1:** Hydrogen-bond geometry (Å, °)

*D*—H⋯*A*	*D*—H	H⋯*A*	*D*⋯*A*	*D*—H⋯*A*
N2—H2⋯O8^i^	0.81	2.28	3.054 (4)	162
O5—H5⋯O6^ii^	0.82	1.82	2.625 (3)	168
C18—H18⋯O5^iii^	0.93	2.58	3.413 (4)	150
C19—H19⋯O6^iv^	0.93	2.48	3.348 (4)	155

Symmetry codes: (i) 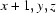; (ii) 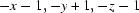; (iii) 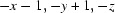; (iv) 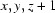.

**Fig. 1.**
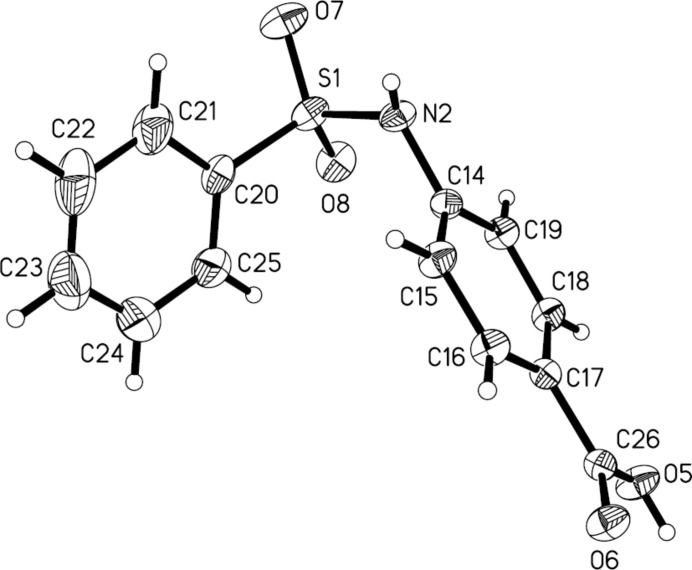
The molecular structure of I with the atom numbering scheme. Displacement ellipsoids are drawn at 30% probability level. H atoms are presented as a small spheres of arbitrary radius.

**Fig. 2.**
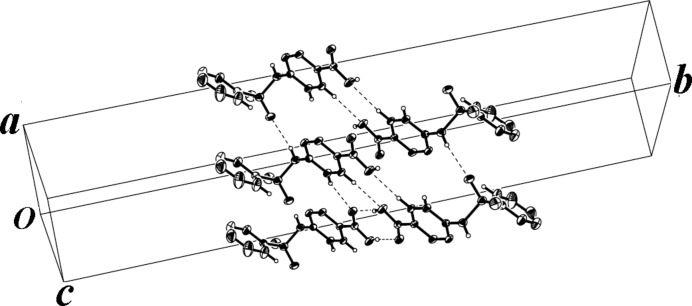
A partial packing diagram of the title compound. Hydrogen bonds are shown as dashed lines and H atoms not involved in hydrogen bonding omitted for clarity.

## supplementary crystallographic information

### Comment

Benzene sulfonamide derivative have shown antimalarial (Parai *et al.*, 2008), carbonic anhydrase inhibitors (Innocenti *et al.*, 2004), anti*HIV* (Selvam *et al.*, 2001) and antiinflamatory (Rathish *et al.*, 2009) activities. In continuation of synthesis and structural studies of different benzene sulfonamide derivative (Khan *et al.*, 2009; Arshad *et al.*, 2009), we report here the molecular and crystal structures of title compound. The molecular structure of the title compound, I, is illustrated in Fig. 1. In I, phenyl and *p*–aminobenzoic acid moieties are connected through the SO_2_ group. The structure of I is comparable with 4–(tosylamino)benzoic acid, (Nan & Xing, 2006). The dihedral angle between the planes of the benzene ring and the carboxyl substituent group is 6.7 (4)°. The two aromatic rings (C20–C25 and C14–C19) are inclined at 45.36 (15)° to one another. The torsion angle C14—N2—S1—C20 in the central part of the molecule is 70 (1)°.

In the crystal, adjacent molecules are linked *via* intermolecular classical N—H···O and O—H···O and non–classical C—H···O hydrogen bonds (Tab. 1, Fig. 2), which stabilize the crystal structure.

### Experimental

The 4–amino benzoic acid (1 g, 7.3 mmol) was dissolved in distilled water (10 ml). The pH of the solution was adjusted at 8–9 using 1*M* Na_2_CO_3_. Benzene sulfonylchloride (1.29 g, 7.3 mmol) was added to the above solution and stirred at room temperature until all the suspended benzene sulfonyl chloride was consumed. On completion of the reaction the pH was adjusted 1–2, using 1*N* HCl acid. The precipitate obtained was filtered, washed with distilled water, dried and recrystalized in methanol to yield colourless crystals.

### Refinement

All H atoms were positioned geometrically an refined using a riding model with C—H = 0.93Å and *U*_iso_(H) = 1.2*U*_eq_(C) for aromatic, O—H = 0.82Å and *U*_iso_(H) = 1.5*U*_eq_(O) for the OH group and N—H = 0.81Å and *U*_iso_(H) = 1.2*U*_eq_(N) for the NH group.

### Crystal data

**Table:**

C_13_H_11_NO_4_S	*F*(000) = 576
*M**_r_* = 277.30	*D*_x_ = 1.433 Mg m^−^^3^
Monoclinic, *P*2_1_/*c*	Mo *K*α radiation, λ = 0.71073 Å
Hall symbol: -P 2ybc	Cell parameters from 5008 reflections
*a* = 5.2050 (3) Å	θ = 3.0–25°
*b* = 37.726 (2) Å	µ = 0.26 mm^−^^1^
*c* = 7.3781 (4) Å	*T* = 295 K
β = 117.510 (3)°	Block, colourless
*V* = 1284.98 (13) Å^3^	0.26 × 0.21 × 0.19 mm
*Z* = 4	

### Data collection

**Table:**

Bruker CCD diffractometer	3185 independent reflections
Radiation source: fine–focus sealed tube	2633 reflections with *I* > 2σ(*I*)
graphite	*R*_int_ = 0.025
φ and ω scans	θ_max_ = 28.4°, θ_min_ = 1.1°
Absorption correction: multi-scan (*SADABS*; Sheldrick, 1996)	*h* = −4→6
*T*_min_ = 0.935, *T*_max_ = 0.958	*k* = −50→45
13550 measured reflections	*l* = −9→9

### Refinement

**Table:**

Refinement on *F*^2^	Primary atom site location: structure-invariant direct methods
Least-squares matrix: full	Secondary atom site location: difference Fourier map
*R*[*F*^2^ > 2σ(*F*^2^)] = 0.065	Hydrogen site location: inferred from neighbouring sites
*wR*(*F*^2^) = 0.199	H-atom parameters constrained
*S* = 1.10	*w* = 1/[σ^2^(*F*_o_^2^) + (0.0878*P*)^2^ + 1.4857*P*] where *P* = (*F*_o_^2^ + 2*F*_c_^2^)/3
3185 reflections	(Δ/σ)_max_ < 0.001
172 parameters	Δρ_max_ = 0.38 e Å^−^^3^
0 restraints	Δρ_min_ = −0.36 e Å^−^^3^

### Special details

**Table:**

Geometry. All s.u.'s (except the s.u. in the dihedral angle between two l.s. planes) are estimated using the full covariance matrix. The cell s.u.'s are taken into account individually in the estimation of s.u.'s in distances, angles and torsion angles; correlations between s.u.'s in cell parameters are only used when they are defined by crystal symmetry. An approximate (isotropic) treatment of cell s.u.'s is used for estimating s.u.'s involving l.s. planes.
Refinement. Refinement of *F*^2^ against ALL reflections. The weighted *R*–factor *wR* and goodness of fit *S* are based on *F*^2^, conventional *R*–factors *R* are based on *F*, with *F* set to zero for negative *F*^2^. The threshold expression of *F*^2^ > σ(*F*^2^) is used only for calculating *R*–factors(gt) *etc*. and is not relevant to the choice of reflections for refinement. *R*–factors based on *F*^2^ are statistically about twice as large as those based on *F*, and *R*–factors based on ALL data will be even larger.

### Fractional atomic coordinates and isotropic or equivalent isotropic displacement parameters (Å^2^)

**Table:**

	*x*	*y*	*z*	*U*_iso_*/*U*_eq_	
S1	0.27263 (17)	0.36345 (2)	0.53929 (12)	0.0443 (3)	
O5	−0.4876 (5)	0.48980 (7)	−0.2675 (3)	0.0496 (6)	
H5	−0.5699	0.5003	−0.3768	0.074*	
O6	−0.2171 (5)	0.47077 (6)	−0.4087 (3)	0.0464 (5)	
O7	0.4834 (6)	0.35320 (8)	0.7386 (4)	0.0693 (8)	
O8	−0.0109 (5)	0.37334 (7)	0.5034 (4)	0.0557 (6)	
N2	0.4098 (5)	0.39697 (7)	0.4738 (4)	0.0408 (6)	
H2	0.5771	0.3942	0.5010	0.049*	
C14	0.2360 (6)	0.41529 (7)	0.2848 (4)	0.0344 (6)	
C15	0.2845 (6)	0.41056 (8)	0.1173 (5)	0.0411 (7)	
H15	0.4269	0.3949	0.1242	0.049*	
C16	0.1192 (6)	0.42930 (8)	−0.0615 (5)	0.0406 (6)	
H16	0.1543	0.4267	−0.1735	0.049*	
C17	−0.0980 (5)	0.45182 (7)	−0.0728 (4)	0.0319 (5)	
C18	−0.1453 (6)	0.45608 (8)	0.0961 (4)	0.0375 (6)	
H18	−0.2921	0.4710	0.0884	0.045*	
C19	0.0239 (6)	0.43826 (8)	0.2754 (4)	0.0386 (6)	
H19	−0.0049	0.4417	0.3895	0.046*	
C20	0.2370 (7)	0.32889 (8)	0.3691 (5)	0.0456 (7)	
C21	0.4682 (10)	0.30652 (11)	0.4110 (8)	0.0731 (12)	
H21	0.6402	0.3090	0.5314	0.088*	
C22	0.4401 (13)	0.28042 (13)	0.2715 (11)	0.0938 (18)	
H22	0.5934	0.2650	0.2995	0.113*	
C23	0.1881 (15)	0.27699 (14)	0.0918 (10)	0.0938 (17)	
H23	0.1703	0.2594	−0.0015	0.113*	
C24	−0.0327 (15)	0.29943 (14)	0.0524 (9)	0.1009 (19)	
H24	−0.2033	0.2972	−0.0692	0.121*	
C25	−0.0115 (10)	0.32565 (11)	0.1883 (7)	0.0724 (12)	
H25	−0.1653	0.3411	0.1574	0.087*	
C26	−0.2763 (6)	0.47182 (7)	−0.2629 (4)	0.0340 (6)	

### Atomic displacement parameters (Å^2^)

**Table:**

	*U*^11^	*U*^22^	*U*^33^	*U*^12^	*U*^13^	*U*^23^
S1	0.0400 (4)	0.0527 (5)	0.0429 (4)	0.0102 (3)	0.0214 (3)	0.0115 (3)
O5	0.0442 (12)	0.0658 (15)	0.0422 (11)	0.0254 (11)	0.0228 (10)	0.0118 (10)
O6	0.0505 (12)	0.0567 (13)	0.0380 (11)	0.0155 (10)	0.0255 (10)	0.0054 (9)
O7	0.0665 (17)	0.087 (2)	0.0466 (14)	0.0161 (15)	0.0193 (13)	0.0233 (13)
O8	0.0467 (13)	0.0653 (15)	0.0681 (16)	0.0086 (11)	0.0376 (12)	0.0058 (12)
N2	0.0281 (11)	0.0490 (14)	0.0410 (13)	0.0053 (10)	0.0121 (10)	0.0076 (10)
C14	0.0279 (12)	0.0365 (14)	0.0357 (13)	0.0003 (10)	0.0122 (10)	0.0004 (10)
C15	0.0350 (14)	0.0452 (16)	0.0460 (15)	0.0146 (12)	0.0210 (12)	0.0041 (12)
C16	0.0410 (15)	0.0483 (16)	0.0395 (14)	0.0095 (12)	0.0247 (12)	0.0008 (12)
C17	0.0277 (12)	0.0343 (13)	0.0342 (13)	0.0010 (10)	0.0146 (10)	−0.0026 (10)
C18	0.0361 (14)	0.0393 (14)	0.0421 (14)	0.0095 (11)	0.0223 (12)	0.0009 (11)
C19	0.0416 (15)	0.0437 (15)	0.0360 (14)	0.0073 (12)	0.0226 (12)	−0.0003 (11)
C20	0.0475 (17)	0.0407 (15)	0.0560 (18)	0.0068 (13)	0.0303 (15)	0.0121 (13)
C21	0.054 (2)	0.058 (2)	0.108 (4)	0.0152 (18)	0.038 (2)	0.003 (2)
C22	0.089 (4)	0.059 (3)	0.156 (6)	0.019 (2)	0.076 (4)	−0.001 (3)
C23	0.130 (5)	0.066 (3)	0.101 (4)	0.008 (3)	0.067 (4)	−0.011 (3)
C24	0.122 (5)	0.076 (3)	0.074 (3)	0.019 (3)	0.019 (3)	−0.015 (3)
C25	0.074 (3)	0.060 (2)	0.066 (2)	0.022 (2)	0.017 (2)	0.0007 (19)
C26	0.0307 (13)	0.0361 (13)	0.0346 (13)	0.0023 (10)	0.0146 (10)	−0.0031 (10)

### Geometric parameters (Å, °)

**Table:**

S1—O7	1.423 (3)	C17—C26	1.482 (4)
S1—O8	1.423 (2)	C18—C19	1.379 (4)
S1—N2	1.632 (3)	C18—H18	0.9300
S1—C20	1.760 (4)	C19—H19	0.9300
O5—C26	1.279 (3)	C20—C25	1.368 (5)
O5—H5	0.8186	C20—C21	1.383 (5)
O6—C26	1.249 (3)	C21—C22	1.383 (7)
N2—C14	1.440 (3)	C21—H21	0.9300
N2—H2	0.8048	C22—C23	1.374 (8)
C14—C19	1.380 (4)	C22—H22	0.9300
C14—C15	1.382 (4)	C23—C24	1.347 (8)
C15—C16	1.390 (4)	C23—H23	0.9300
C15—H15	0.9300	C24—C25	1.376 (7)
C16—C17	1.386 (4)	C24—H24	0.9300
C16—H16	0.9300	C25—H25	0.9300
C17—C18	1.386 (4)		
			
O7—S1—O8	120.12 (17)	C14—C19—C18	119.7 (3)
O7—S1—N2	106.43 (16)	C14—C19—H19	120.1
O8—S1—N2	107.42 (14)	C18—C19—H19	120.1
O7—S1—C20	108.24 (17)	C25—C20—C21	119.8 (4)
O8—S1—C20	107.64 (16)	C25—C20—S1	120.2 (3)
N2—S1—C20	106.21 (14)	C21—C20—S1	119.9 (3)
C26—O5—H5	109.5	C20—C21—C22	119.1 (5)
C14—N2—S1	119.58 (19)	C20—C21—H21	120.4
C14—N2—H2	115.3	C22—C21—H21	120.5
S1—N2—H2	113.4	C23—C22—C21	120.7 (5)
C19—C14—C15	120.5 (3)	C23—C22—H22	119.6
C19—C14—N2	118.7 (3)	C21—C22—H22	119.6
C15—C14—N2	120.7 (2)	C24—C23—C22	119.2 (5)
C14—C15—C16	119.6 (3)	C24—C23—H23	120.4
C14—C15—H15	120.2	C22—C23—H23	120.4
C16—C15—H15	120.2	C23—C24—C25	121.4 (5)
C17—C16—C15	119.9 (3)	C23—C24—H24	119.3
C17—C16—H16	120.0	C25—C24—H24	119.3
C15—C16—H16	120.0	C20—C25—C24	119.7 (4)
C18—C17—C16	119.7 (3)	C20—C25—H25	120.1
C18—C17—C26	119.9 (2)	C24—C25—H25	120.1
C16—C17—C26	120.4 (2)	O6—C26—O5	123.0 (3)
C19—C18—C17	120.4 (3)	O6—C26—C17	120.2 (2)
C19—C18—H18	119.8	O5—C26—C17	116.8 (2)
C17—C18—H18	119.8		

### Hydrogen-bond geometry (Å, °)

**Table:**

*D*—H···*A*	*D*—H	H···*A*	*D*···*A*	*D*—H···*A*
N2—H2···O8^i^	0.81	2.28	3.054 (4)	162
O5—H5···O6^ii^	0.82	1.82	2.625 (3)	168
C18—H18···O5^iii^	0.93	2.58	3.413 (4)	150
C19—H19···O6^iv^	0.93	2.48	3.348 (4)	155

Symmetry codes: (i) *x*+1, *y*, *z*; (ii) −*x*−1, −*y*+1, −*z*−1; (iii) −*x*−1, −*y*+1, −*z*; (iv) *x*, *y*, *z*+1.
